# Ultrasound stimulation of the vagus nerve as a treatment modality for anxiety

**DOI:** 10.3389/fpsyt.2024.1376140

**Published:** 2024-10-02

**Authors:** Michell Goyal, Ravi Goyal, Joseph L. Sanguinetti

**Affiliations:** ^1^ Department of Physiology, University of Arizona, Tucson, AZ, United States; ^2^ Department of Obstetrics and Gynecology, University of Arizona, Tucson, AZ, United States; ^3^ Center for Consciousness Studies, University of Arizona, Tucson, AZ, United States

**Keywords:** ultrasound stimulation, vagus nerve, anxiety, vagus nerve stimulation, electrical vagus nerve stimulation

## Abstract

Anxiety is an increasingly prevalent mental disorder, causing widespread hardship and interfering with society’s economic progression. Standard treatments include various talk therapies with poor prognoses or drug interventions with complex side effects, both introducing unnecessary burdens to patients. To remedy this, non-invasive ultrasound stimulation to the vagus nerve is a novel, low-cost treatment that is showing promise. Although vagus nerve stimulation is already approved for epilepsy and other conditions, it requires regular maintenance. In contrast, studies using non-invasive ultrasound stimulation have shown preliminary positive results in affecting vagal activity with minimal drawbacks. This review covers a variety of studies investigating the effects of ultrasound stimulation on the vagus nerve. With rising levels of anxiety with each generation, there is a pressing need for more innovative and diverse treatments with fewer costs and more benefits.

## 1 Introduction

According to the Diagnostic and Statistical Manual of Mental Disorders Fifth Edition, an anxiety disorder is characterized by excessive, distressing, and persistent anxiety or maladaptive behaviors to avoid the anxiety-provoking entity or situation (1). Some examples of anxiety disorders include social anxiety, generalized anxiety disorder, specific phobias, panic disorder (PD), and many others. The National Alliance on Mental Illness reports that the annual prevelance of US adults with an anxiety disorder is almost 20%, one in five adults, which is higher than any other mental health condition (2). Generalized anxiety disorder is reported to affect almost 3.1% of US adults, although only 43.2% of these individuals receive treatment (3). These staggering numbers indicate the severity of this issue. Mental disorders can also cause more than just emotional hardships. They account for greater economic costs than chronic somatic diseases like cancer or diabetes (4). In 2020, the global direct economic costs of a mental disorder were about $6.5 trillion (5). This is because the overall costs for mental disorders can include more than just “direct costs” like visits to a clinic or hospital, medication, and therapy. There are also many “indirect costs” such as income losses due to disability and lost production from work absence. Problems like this are evidence that mental disorders like anxiety are not only an emotional tragedy but also devastating for the entire community.

Despite these facts, our knowledge to treat this disorder is limited. It is important to note that the treatment gap for mental and substance-use disorders is also greater than for any other health sector (6). Current treatment options are not always effective in reducing one’s anxiety levels and may take significant time to demonstrate an effect. One type of treatment is psychotherapy, also known as “talk therapy.” An example of psychotherapy is cognitive behavioral therapy. This is especially helpful for people with anxiety disorders, as it teaches different ways of thinking, behaving, and reacting to certain triggers. It focuses on identifying and challenging unhelpful, negative, and/or distorted thoughts underlying one’s anxiety. In contrast, exposure therapy works by confronting the anxiety-inducing object or activity to reduce fear of it. Although these methods have proven to work, they do require time to learn, take effect, and are costly. Before therapy, however, medications are usually prescribed as the first and second lines of treatment. To combat anxiety, anti-anxiety medicines like benzodiazepines and anti-depressants are used. The problem with medications, though, is that there are often adverse effects, such as suicidal thoughts. They can also be addictive and have harsh withdrawal symptoms (7). Medications also require time to have measurable effects. Moreover, these treatments have little effect when it comes to chronic and treatment-resistant anxiety. However, a new method is being developed to treat treatment-resistant and chronic anxiety that is both non-invasive and fast-acting. This treatment is non-invasive ultrasound-mediated stimulation of the vagus nerve (VUS). Notably, invasive vagus nerve stimulation (iVNS) to treat various disorders is already used as a therapeutic modality for various disorders. Furthermore, transcutaneous vagus nerve stimulation is an alternative non-invasive treatment option being investigated. However, that is not the focus of the present review and has been reviewed elsewhere (8).

## 2 Invasive vagus nerve stimulation

The vagus nerve is also known as the “wandering nerve” because it spreads widely across the body. It begins in the brain and extends through the neck and chest to the abdomen. The nerve also has cardiac, esophageal, and pulmonary branches. It serves to transmit motor impulses, slow heart rate, and control involuntary muscles in the esophagus, stomach, gallbladder, pancreas, and small intestine (9). Notably, electrical iVNS has already been Food and Drug Administration (FDA)–approved for the treatment of epilepsy, treatment-resistant depression, and ischemic stroke (10, 11). Of note, the first implantable stimulator was developed in 1988 for epilepsy. The small battery-operated device has an electrode wire that wraps around the left cervical portion of the vagus nerve, which stimulates sensory fibers that communicate with target regions of the brain to improve mood (12). About 40% of patients using iVNS had a 50% reduction in seizures after 2–3 years of treatment (13). However, the battery-operated device required battery replacement and had side effects such as dysphonia, hoarseness, and cough. A non-implant transcutaneous VNS method was studied by the Chinese Academy of Chinese Medical Sciences, which found a 75% reduction in the frequency of seizures in 41% of the patients (13, 14). Side effects were also minimal. A study involving iVNS to treat depression was conducted in which 53.1% of patients had a 50% decrease in the Hamilton Rating Scale for Depression, whereas 38.9% fulfilled the remission criteria (13). In a separate study, iVNS had a lower mortality rate and an anti-suicidal effect as compared to electro-convulsion therapy (12, 15–17).

iVNS is already being used successfully to treat such disorders; however, its use for anxiety is still limited. In one study, rats were given iVNS after auditory fear conditioning along with extinction training. Half of the rats were given iVNS, whereas the other half received sham stimulation. As a result, iVNS promoted the generalization of fear extinction. In the same report, rats showed increased time spent in the open arms of an elevated plus maze after VNS administration, which is indicative of reduced anxiety (11). In a pilot study for treatment-resistant anxiety disorders, 11 outpatients with obsessive-compulsive disorder (OCD), PD, and posttraumatic stress disorder were given iVNS along with psychotropic medications for 12 weeks. Nine of the 11 patients were able to finish the study. There was a 50% or greater improvement on the Hamilton Anxiety Scale for three of the nine patients and a 25% or greater improvement on the Yale-Brown Obsessive-Compulsive Scale for patients with OCD (18). This is evidence for the potential of long-term improvement and a reduction of anxiety due to VNS.

## 3 Ultrasound stimulation mechanism of action

Although iVNS is already being utilized as a treatment, more non-invasive methods are needed. VUS appears to be a plausible alternative because ultrasound is used frequently in the medical field and is already widely available. It is most commonly used in imaging during pregnancy to monitor the baby’s health. It is also used to diagnose gallbladder disease, evaluate blood flow, and assess joint inflammation. Instead of using ultrasound for imaging, focused ultrasound can be used, such as in these studies, for neuromodulation. In contrast to VUS, iVNS involves implanting “cuff electrodes” onto the nerve that requires surgery and have higher chances of causing nerve damage (19, 20). In 2014, VUS was studied as an alternative to iVNS (21). Specifically, Juan et al. investigated acoustic neuromodulation using low-intensity focused ultrasound to stimulate or inhibit neural structures. The preliminary study concluded that acoustic neuromodulation using ultrasound does affect vagus nerve function (21). In another study, VUS in rats was observed to reduce tumor necrosis factor–alpha (TNF-a) levels that are linked to chronic inflammatory diseases. Importantly, iVNS is also associated with a reduction in TNF-a level. (20). Both studies suggest that VUS may have similar effects as iVNS and can be an effective non-invasive alternative.

The most common method of ultrasound stimulation currently being investigated is transcranial ultrasound stimulation. In this, pulses of ultrasonic waves are focused at various regions of the brain. This modulates the brain areas with either inhibitory or excitatory effects, depending on the parameters used for stimulation, including pulse frequency, duration, and others (22).

Ultrasound stimulation modulates the nervous system’s activity via either thermal or mechanical effects. In the thermal effects of ultrasound stimulation, the energy of the waves increases the temperature of the focused area of nervous tissue, in turn affecting the waveforms of the neuronal electrical potentials. In its mechanical effects, it has been shown that the radiation effects of ultrasonic waves may cause a temporary displacement of the cell membrane. This affects stretch-activated ion channels in the membrane, causing a change in the resting potential of the cell. Thus, depending on the intensity of the stimulation, it can cause either inhibitory or excitatory effects on the area of modulation because the excitability of the cell is altered (23). This is likely the case at low intensities, where thermal energy plays a lesser role. Conversely, the thermal effects of high-intensity ultrasound stimulation have been shown to override the mechanical effects, possibly due to increased temperatures disabling ion channels (24). These studies suggest that high-intensity stimulation mainly causes inhibitory effects by ultrasound stimulation to neuronal cells, whereas low-intensity stimulation causes excitatory effects (21). However, the cutoff for low versus high intensities needs to be more clearly defined, and the effects of ultrasound stimulation may differ in different cell types (23).

## 4 Role of vagus nerve in anxiety

The vagus nerve is the main mechanistic component of the parasympathetic nervous system (PNS), which opposes the body’s fight-or-flight responses from the sympathetic nervous system (SNS). As previously mentioned, it modulates the responses of many of the body’s vital organs, most importantly the heart. During a fight-or-flight response, the SNS redirects blood flow away from the digestive system and toward the muscles and brain, among other organs. To do this, in part, it increases heart rate. After the threat is gone, the PNS steps in to reverse all actions, calming the heart and restoring the resting state of the body.

Sometimes, the PNS is unable to completely calm the body, causing the person to feel jittery and “on-edge.” This biological inability can be quantified by examining a person’s heart rate variability (HRV), the measure of variation between heartbeats. Essentially, changes in HRV can be used to determine how quickly the heart can switch between its resting state and an excited state. It has been shown that those with anxiety disorders present with a reduced HRV, which thus indicates impaired vagal function (25). This study also mentions reduced HRV measurements present in those with depression and alcohol dependence, consistent with the known comorbidity of the conditions.

VUS can cause temporary excitatory effects (26), which would be reparative effects of vagal function, as described above. Moreover, the body has a powerful adaptive ability, which is the basis of the traditional “talk therapy” and versions of cognitive behavioral therapy previously explained. We speculate that the body’s adaptiveness to more intensive biological modulation, such as VUS, may be as powerful as current therapies. Therefore, it is logical to assume that VUS, specifically its excitatory effects at low intensities (21), may have the potential to improve vagal function. Thus, it may also improve symptoms of anxiety (and its comorbid conditions) and act as an adjuvant to current conventional therapies. However, further investigation is required.

## 5 Current studies using vagus nerve stimulation

A search on PubMed was conducted with the keywords “vagus nerve” or “vagal nerve” and “ultrasound stimulation” and “non-invasive,” which presented 55 studies. Studies not using non-invasive ultrasound stimulation or those not targeting the vagus nerve or peripheral nerves as one of the targets were excluded from the review. Studies where the main aim was to visualize the vagus nerve, test vagus nerve imaging devices, or design parts of such imaging devices were also excluded. Additionally, studies that were not in English were excluded. An additional Google Search was conducted with the keywords “ultrasound stimulation of the vagus nerve,” which presented 15 studies. The same parameters used in the previous search were used to exclude studies from this search. Additionally, duplicate studies also present in the previous search as well as review articles were disregarded. This produced 11 studies total included in the review.

Notably, the studies reviewed inconsistently reported the parameters used for ultrasound stimulation. Frequency measurements ranged widely from 5,000 Hz to 3.7 MHz. Intensity also ranged between 1.86 W/cm^2^ and 120.63 W/cm^2^. For most studies, the spatial peak pulse average intensity was given which is the average intensity of the ultrasonic pulses administered) (27). Thus, a standardization regarding the frequency, intensity, and pulses to define low, moderate, and severe stimulation is required.

Until now, no studies have been conducted on the direct relationship between VUS and its effects on anxiety. However, multiple studies have investigated the effects of VUS on disorders other than anxiety (**Table 1**). Many of these reports present consistent results, including a reduction in inflammatory markers, activation of anti-inflammatory pathways, and physiological effects commonly associated with stimulation of the PNS, such as reduced heart rate and blood pressure. Interestingly, one study also presented improved anhedonic-like behavior in mice modeled for Parkinson’s disease (35). Anhedonia is a symptom of depressive disorders, which, as mentioned, is highly comorbid with anxiety disorders. Therefore, VUS has the potential to become a non-invasive, low-risk treatment for neuropsychological illnesses, which currently have limited options.

**Table 1:** Studies demonstrating use of VUS in different disorders.

Reference	Model	Target	Parameters*	findings
(26)	Mice	Hippocampus and cervical VN	Frequency 1 = 300 kHz and frequency 2 = 1.0 MHz	Ultrasound VNS evokes APs
(20)	Rats	VN	Frequency = 250 kHz and I_SPPA_ = 3 W/cm^2^	Reduced inflammatory markers
(28)	Humans	Cervical VN and spleen	I_sppa_ = 1.86 W/cm^2^	Reduced heart rate and reduced inflammatory markers
(29)	Rabbits	VN	Frequency = 3.7 MHz and I_sppa_ = 18–87 W/cm^2^	Reduced heart rate and reduced blood pressure
(21)	Rats	Cervical VN	Frequency = 1.1 MHz and I_sppa_ = 13.6–93.4 W/cm^2^	Proportional relationship between intensity and inhibition
(30)	Mice	Peripheral nerves in the liver and spleen	Frequency = 1.1 MHz and I < 35 W/cm^2^	Reduced inflammatory responses
(31)	Rats	VN	Frequency = 1.71 MHz	Reduced inflammatory markers
(32)	Humans	Cervical VN	Frequency = 5,000 Hz and amplitude = 24 V	Increased attention/alertness
(33)	Rats and *in vitro*	Various peripheral nerves and ganglia	Frequency = 1.1 MHz	Activation of peripheral neurons and activation of anti-inflammatory pathway
(34)	Mice	VN	Frequency = 0.5 MHz and I = 19.3–120.63 W/cm^2^	Improved cardiac function and reduced inflammatory markers
(35)	Rats	Celiac plexus	Frequency = 2.5 MHz	Improved anhedonic-like behavior

*Parameters given were inconsistent between studies.

## 6 Safety considerations

VNS is currently FDA-approved for the treatment of seizures, depression, and stroke and is not known to produce any major adverse effects. Therapeutic ultrasound is currently being used for musculoskeletal pathologies, atrial ablation, accelerating healing processes, and treatment of soft tissue tumors. Ultrasound therapy devices are also available for consumer purchase and are safe to use without medical experts’ supervision. Although focused ultrasound can be used to cause lesions, this level of ultrasound is not available for consumer purchase.

## 7 Conclusion

VUS has been proven to enhance the effects of the PNS. VUS has also been proven to be a non-invasive alternative to the more common iVNS. A review of current literature shows promising results that can be utilized to improve symptoms of psychological disorders. With rising levels of anxiety in each generation, the need for more options has increased dramatically. There are currently limited methods to reduce one’s anxiety, and they often introduce more costs than benefits. In addition, medications and common treatments may have adverse effects (7). Importantly, ultrasound stimulation is already a proven therapy that is well investigated by authoritative bodies for several disorders. Thus, for anxiety disorders as well as other neuropsychological conditions, VUS will be an additional therapeutic option, which may significantly change how we treat anxiety, and further investigation is needed to make it a standard therapy.

## 8 Future research

Studies investigating the relationship between VUS or ultrasound stimulation in general and its effects on psychological disorders are sparse. Efforts to bridge these gaps would provide clarity to both the mechanism of action of ultrasound therapy as well as the biological intricacies of these illnesses.
